# PB.9. Randomised controlled trial of stereotactic 11G vacuum-assisted core biopsy for diagnosis and management of malignant microcalcification

**DOI:** 10.1186/bcr3741

**Published:** 2014-11-03

**Authors:** S Bundred, A Maxwell, J Morris, J Harake, S Whiteside, J Zhou, N Bundred

**Affiliations:** 1 University Hospital South Manchester, Manchester, UK; 2 University of Manchester, UK; 3 Royal Bolton NHS Foundation Trust, Bolton, UK

## Introduction

Vacuum-assisted biopsy (VAB) may improve accuracy of diagnosis of malignant microcalcification (MM) compared with 14G core biopsy (SCNB), thus reducing requirements for further surgical procedures.

## Methods

VAB and SCNB were compared in a randomised controlled trial (1:1) in first-line diagnosis of MM and subsequent surgical outcomes in two UK breast screening units. Participants gave written informed consent prior to randomisation. Exclusions included bleeding diathesis or comorbidity precluding surgery. SCNB was performed by advanced radiography practitioners and VAB by radiologists. Quality-of-life (QOL) was assessed using the EORTC QLQ BR-23 questionnaire pre biopsy, 2, 6 and 12 months post randomisation. Final pathological diagnosis was compared with the initial biopsy result. A total of 110 participants were required to show a 25% improvement in diagnosis with VAB compared with SCNB (90% power).

## Results

PPV for MM biopsy was 30.2%. Groups were evenly matched for age and lesion size (75% <15 mm). Eligibility was assessed for 787 cases; 129 women were randomised. Diagnostic accuracy of VAB was 86% and SCNB was 84%, which precluded any effect on surgical outcomes; 12.5% of VAB cases (8.7% SCNB) upgraded from DCIS at surgery. Following VAB, 44% cases required repeat surgery (29% SCNB). VAB took longer (*P *< 0.001) and yielded more specimens (12 vs. 8) (*P *< 0.01). Three VAB (4.7%) procedures were complicated by severe bleeding. Significant falls in QOL scores for global health (*P *< 0.001) and social functioning (*P *< 0.04) were observed over time for both groups.

## Conclusion

SCNB and VAB were equally accurate in the first-line diagnosis of malignant microcalcification.

